# Early Estimates of Updated 2023–2024 (Monovalent XBB.1.5) COVID-19 Vaccine Effectiveness Against Symptomatic SARS-CoV-2 Infection Attributable to Co-Circulating Omicron Variants Among Immunocompetent Adults — Increasing Community Access to Testing Program, United States, September 2023–January 2024

**DOI:** 10.15585/mmwr.mm7304a2

**Published:** 2024-02-01

**Authors:** Ruth Link-Gelles, Allison Avrich Ciesla, Josephine Mak, Joseph D. Miller, Benjamin J. Silk, Anastasia S. Lambrou, Clinton R. Paden, Philip Shirk, Amadea Britton, Zachary R. Smith, Katherine E. Fleming-Dutra

**Affiliations:** ^1^National Center for Immunization and Respiratory Diseases, CDC; ^2^Eagle Health Analytics, San Antonio, Texas; ^3^Office of Readiness and Response, CDC.

**Summary**

**What is already known about this topic?**

In September 2023, CDC’s Advisory Committee on Immunization Practices recommended updated 2023–2024 (monovalent XBB.1.5) COVID-19 vaccination for all persons aged ≥6 months to prevent COVID-19, including severe disease. Many variants co-circulated during fall 2023; the JN.1 lineage became predominant in January 2024. Few estimates of updated 2023–2024 vaccine effectiveness (VE) are available.

**What is added by this report?**

Receipt of updated COVID-19 vaccine provided approximately 54% increased protection against symptomatic SARS-CoV-2 infection compared with no receipt of updated vaccine. Vaccination provides protection against JN.1 and other circulating lineages.

**What are the implications for public health practice?**

All persons aged ≥6 months should receive updated 2023–2024 COVID-19 vaccine. CDC will continue monitoring COVID-19 VE, including against severe disease and for expected waning.

## Abstract

On September 12, 2023, CDC’s Advisory Committee on Immunization Practices recommended updated 2023–2024 (updated) COVID-19 vaccination with a monovalent XBB.1.5–derived vaccine for all persons aged ≥6 months to prevent COVID-19, including severe disease. During fall 2023, XBB lineages co-circulated with JN.1, an Omicron BA.2.86 lineage that emerged in September 2023. These variants have amino acid substitutions that might increase escape from neutralizing antibodies. XBB lineages predominated through December 2023, when JN.1 became predominant in the United States. Reduction or failure of spike gene (*S*-gene) amplification (i.e., *S*-gene target failure [SGTF]) in real-time reverse transcription–polymerase chain reaction testing is a time-dependent, proxy indicator of JN.1 infection. Data from the Increasing Community Access to Testing SARS-CoV-2 pharmacy testing program were analyzed to estimate updated COVID-19 vaccine effectiveness (VE) (i.e., receipt versus no receipt of updated vaccination) against symptomatic SARS-CoV-2 infection, including by SGTF result. Among 9,222 total eligible tests, overall VE among adults aged ≥18 years was 54% (95% CI = 46%–60%) at a median of 52 days after vaccination. Among 2,199 tests performed at a laboratory with SGTF testing, VE 60–119 days after vaccination was 49% (95% CI = 19%–68%) among tests exhibiting SGTF and 60% (95% CI = 35%–75%) among tests without SGTF. Updated COVID-19 vaccines provide protection against symptomatic infection, including against currently circulating lineages. CDC will continue monitoring VE, including for expected waning and against severe disease. All persons aged ≥6 months should receive an updated COVID-19 vaccine dose.

## Introduction

On September 12, 2023, CDC’s Advisory Committee on Immunization Practices recommended that all persons aged ≥6 months receive the updated 2023–2024 (updated) monovalent COVID-19 vaccine (*1*). Most persons aged ≥5 years are recommended to receive 1 updated dose. These vaccines contain a component from the SARS-CoV-2 Omicron XBB.1.5 lineage and unlike previous COVID-19 vaccines, do not contain the ancestral SARS-CoV-2 strain. During the period of analysis, XBB lineages predominated early, many with evolutionarily advantageous amino acid changes in the spike gene (*S*-gene). In September 2023, the divergent JN.1 lineage was detected in the United States. JN.1 has more than 30 mutations in the spike protein compared with XBB.1.5, including a change (L455S) similar to one found in circulating XBB lineages (L455F).* JN.1 accounted for 69% (range = 65%–73%) of SARS-CoV-2 infections nationally by the 2-week period ending January 6, 2024.^†^ Results of spike gene (*S*-gene) amplification in real-time reverse transcription–polymerase chain reaction (RT-PCR) can be used to distinguish certain SARS-CoV-2 lineages over time (*2*). Detection of *S*-gene target presence (SGTP) by a widely used commercial test was noted in most lineages that circulated in 2023, including XBB lineages,^§^ whereas *S*-gene target failure (SGTF), resulting from a mutation in the *S*-gene, is detected in JN.1 and other BA.2.86 lineages.^¶^ Vaccine effectiveness (VE) of receipt of updated COVID-19 vaccine in preventing symptomatic SARS-CoV-2 infection was assessed in adults aged ≥18 years, by time since dose and by SGTF and SGTP as a proxy for likely JN.1 versus other lineages. Whereas the goal of the U.S. COVID-19 vaccination program is to prevent severe disease, VE against symptomatic infection can provide useful insights into protection early after introduction of updated vaccines and during the emergence of new lineages, such as JN.1.

## Methods

### Overall Assessment of VE

Increasing Community Access to Testing (ICATT) is a CDC program that provides access to no-cost SARS-CoV-2 testing at pharmacies nationwide to persons who are uninsured,** prioritizing socially vulnerable areas.^††^ ICATT VE methods have been described^§§^ (*3*). Tests conducted at participating CVS Pharmacy and Walgreen Co. (Walgreens) locations during September 21, 2023–January 14, 2024, among adults who reported ≥1 symptom of COVID-19 were included in the test-negative design study. For the full analysis, case-patients were persons who received a positive nucleic acid amplification test (NAAT) result; control patients were those who received a negative NAAT result. Tests among persons fulfilling any of the following criteria were excluded from analyses: 1) self-reported immunocompromising condition^¶¶^; 2) reported receipt of Novavax as the most recent dose and reported receipt of <2 total COVID-19 vaccine doses***; 3) reported receipt of a Janssen (Johnson & Johnson) COVID-19 vaccine dose after May 12, 2023^†††^; 4) receipt of the most recent dose <7 days before the date of testing or during September 1–12, 2023; 5) receipt of a COVID-19 vaccine <2 months before date of testing for those who did not receive an updated COVID-19 vaccine dose; or 6) registration for testing with a version of the questionnaire that only reported month and year of the most recent vaccine dose rather than calendar date. In addition, tests from persons reporting receipt of a positive SARS-CoV-2 test result during the preceding 90 days^§§§^ were excluded. Type of most recent vaccine dose (original monovalent, bivalent, or updated monovalent) was determined by the reported date of receipt of the dose.^¶¶¶^

VE against symptomatic disease was calculated by comparing odds of receipt versus nonreceipt of the updated COVID-19 vaccine among case- and control patients. Secondary analyses examined alternative reference groups, including 1) receipt of a bivalent dose and 2) being either unvaccinated or having received only original COVID-19 vaccines. Odds ratios (ORs) were estimated using multivariable logistic regression****; VE was calculated separately based on SGTF or SGTP status as (1 − OR) x 100%.

### Analysis of VE by SGTF and Time Since Vaccination

A subanalysis of VE by SGTF status and time since last dose included RT-PCR tests performed by one pharmacy chain during October 27, 2023–January 12, 2024, and analyzed at a commercial laboratory that used the TaqPath COVID-19 Combo Kit (Thermo Fisher Scientific). Quantitative results were reported as cycle threshold (Ct) values for each of three SARS-CoV-2 gene targets (*S, N*, and *ORF1ab*). Only specimens with Ct values available for both *N* and *ORF1ab* were included in the SGTF subanalysis. SARS-CoV-2–positive specimens with either null or reduced amplification of the *S*-gene (Ct for *S*-gene >4 cycles from the average of *N* and *ORF1ab* Ct values) were considered to have SGTF (*2*,*4*), an indication of a particular deletion in the SARS-CoV-2 spike protein, which currently indicates an infection with BA.2.86, JN.1, and their sublineages. SARS-CoV-2–positive specimens without SGTF were considered to exhibit SGTP, which likely indicates infection with previously dominant XBB.1.5 lineages (Supplementary Figure; https://stacks.cdc.gov/view/cdc/145936).

For the SGTF and SGTP subanalysis, overall VE (regardless of time since dose) and VE during the 7–59 days after an updated dose were not calculated because the emergence of JN.1 parallels time since dose; statistical power for SGTF (likely JN.1) during the 7–59 days was therefore limited. Analyses were conducted using R software (version 4.1.2; R Foundation). This activity was reviewed by CDC, deemed not research, and was conducted consistent with applicable federal law and CDC policy.^††††^

## Results

### Overall VE

Among 9,222 NAAT results for persons with COVID-19–like illness symptoms eligible for the full analysis, 3,295 (36%) were positive for SARS-CoV-2 (Table 1). Among 1,125 persons who had received updated COVID-19 vaccine ≥7 days earlier, more control patients (844; 14%) reported having received the vaccine than did case-patients (281; 9%). Among those who received updated vaccine, the median interval since the last dose was 60 days (IQR = 32–79 days) for case-patients and 51 days (IQR = 28–73) for control patients. Among the 8,097 persons who reported that they had not received an updated vaccine dose, 2,435 (30%) were unvaccinated. Among the remaining 5,662 (70%) who were vaccinated but had not received an updated vaccine dose, the median interval since the last dose was 378 days (IQR = 321–413 days) for case-patients and 363 days (IQR = 254–402 days) for control patients. In the full analysis, VE for persons aged 18–49 years was 57% (95% CI = 48%–65%) and for persons aged ≥50 years was 46% (95% CI = 31%–58%) (Table 2). Overall VE was 58% (95% CI = 48%–65%) among those who received testing 7–59 days after receipt of updated vaccine and 49% (95% CI = 36%–58%) among those who received testing 60–119 days after receipt of updated vaccine.

**TABLE 1:** Characteristics of patients with SARS-CoV-2 tests conducted at national pharmacy testing locations (N = 9,222) — Increasing Community Access to Testing program, United States, September 2023–January 2024

Characteristic	Full analysis (all eligible NAATs), no. (column %)			Subanalysis (eligible TaqPath COVID-19 Combo Kit tests only),* no. (column %)			
	Total no. of tests	SARS-CoV-2–negative (control patients) n = 5,927	SARS-CoV-2–positive (case-patients) n = 3,295	Total no. of tests	SARS-CoV-2–negative (control patients) n = 1,520	SGT presence (likely non-JN.1) n = 421	SGT failure (likely JN.1) n = 258
**All tests (row %)**	**9,222 (100)**	**5,927 (64)**	**3,295 (36)**	**2,199 (100)**	**1,520 (69)**	**421 (19)**	**258 (12)**
**Age group, yrs**							
18–49	**7,155 (78)**	4,673 (79)	2,482 (75)	**1,694 (77)**	1,187 (78)	306 (73)	201 (78)
50–64	**1,547 (17)**	916 (15)	631 (19)	**363 (17)**	238 (16)	88 (21)	37 (14)
≥65	**520 (6)**	338 (6)	182 (6)	**142 (6)**	95 (6)	27 (6)	20 (8)
**Gender**							
Female	**5,581 (61)**	3,656 (62)	1,925 (58)	**1,341 (61)**	966 (64)	233 (55)	142 (55)
Male	**3,586 (39)**	2,228 (38)	1,358 (41)	**836 (38)**	535 (35)	187 (44)	116 (45)
Other	**55 (1)**	43 (1)	12 (0.4)	**22 (1)**	21 (1)	1 (0.2)	0 (—)
**Race and ethnicity^†^**							
Black or African American	**1,465 (16)**	1,002 (17)	463 (14)	**273 (12)**	223 (15)	27 (6)	23 (9)
White	**3,578 (39)**	2,277 (38)	1,301 (39)	**1,003 (46)**	622 (41)	245 (58)	136 (53)
Hispanic or Latino	**2,662 (29)**	1,717 (29)	945 (29)	**512 (23)**	400 (26)	68 (16)	44 (17)
Other	**815 (9)**	487 (8)	328 (10)	**226 (10)**	147 (10)	52 (12)	27 (10)
Unknown	**702 (8)**	444 (7)	258 (8)	**185 (8)**	128 (8)	29 (7)	28 (11)
**HHS testing site region^§^**							
1	**316 (3)**	201 (3)	115 (3)	**143 (7)**	96 (6)	24 (6)	23 (9)
2	**999 (11)**	463 (8)	536 (16)	**368 (17)**	151 (10)	126 (30)	91 (35)
3	**519 (6)**	351 (6)	168 (5)	**135 (6)**	86 (6)	35 (8)	14 (5)
4	**1,775 (19)**	1,260 (21)	515 (16)	**413 (19)**	399 (22)	39 (9)	35 (14)
5	**1,286 (14)**	823 (14)	463 (14)	**298 (14)**	213 (14)	59 (14)	26 (10)
6	**2,048 (22)**	1,403 (24)	645 (20)	**358 (16)**	327 (22)	20 (5)	11 (4)
7	**225 (2)**	141 (2)	84 (3)	**29 (1)**	25 (2)	3 (1)	1 (0.4)
8	**207 (2)**	136 (2)	71 (2)	**36 (2)**	27 (2)	6 (1)	3 (1)
9	**1,593 (17)**	971 (16)	622 (19)	**396 (18)**	236 (16)	107 (25)	53 (21)
10	**254 (3)**	178 (3)	76 (2)	**23 (1)**	20 (1)	2 (0.5)	1 (.04)
**SVI, mean (SD)^¶^**	**0.6 (0.3)**	0.6 (0.3)	0.6 (0.3)	**0.5 (0.3)**	0.5 (0.3)	0.5 (0.3)	0.5 (0.3)
**Self-reported history of SARS-CoV-2–positive test result**							
None	**3,699 (40)**	2,267 (38)	1,432 (43)	**807 (37)**	545 (36)	169 (40)	93 (36)
Positive >90 days before current test	**5,523 (60)**	3,660 (62)	1,863 (57)	**1,392 (63)**	975 (64)	252 (60)	165 (64)
**SARS-CoV-2 test type**							
Rapid NAAT**	**5,338 (58)**	3,438 (58)	1,900 (58)	**NA**	NA	NA	NA
Laboratory-based NAAT^††^	**3,884 (42)**	2,489 (42)	1,395 (42)	**2,199 (100)**	1,582 (100)	421 (100)	258 (100)
**At least one self-reported chronic underlying condition**							
No	**5,966 (65)**	3,844 (65)	2,122 (64)	**1,389 (63)**	955 (63)	280 (67)	154 (60)
Yes	**3,256 (35)**	2,083 (35)	1,173 (36)	**810 (37)**	565 (37)	141 (33)	104 (40)
**Self-reported most recent COVID-19 vaccine dose received before test date^§§,¶¶^**							
Unvaccinated	**2,435 (26)**	1,705 (29)	730 (22)	**430 (20)**	333 (22)	68 (16)	29 (11)
Original monovalent	**4,493 (49)**	2,669 (45)	1,824 (55)	**1,140 (52)**	749 (49)	245 (58)	146 (57)
Bivalent	**1,169 (13)**	709 (12)	460 (14)	**402 (18)**	264 (17)	85 (20)	53 (21)
Updated dose, ≥7 days earlier	**1,125 (12)**	844 (14)	281 (9)	**227 (10)**	174 (11)	23 (5)	30 (12)
Updated dose, 7–59 days earlier	**634 (7)**	494 (8)	140 (4)	**NA**	NA	NA	NA
Updated dose, 60–119 days earlier	**491 (5)**	350 (6)	141 (4)	**227 (10)**	174 (11)	23 (5)	30 (12)
**Updated dose product manufacturer^¶¶^**							
Moderna	**472 (5)**	356 (6)	116 (4)	**91 (4)**	72 (5)	7 (2)	12 (5)
Novavax	**49 (1)**	43 (1)	6 (0.2)	**5 (0.2)**	4 (0.3)	0 (—)	1 (0.4)
Pfizer-BioNTech	**604 (7)**	445 (8)	159 (5)	**131 (6)**	98 (6)	16 (4)	17 (7)
None	**8,097 (88)**	5,083 (86)	3,014 (91)	**1,972 (90)**	1,346 (89)	398 (95)	228 (88)
**Self-reported total no. of COVID-19 vaccine doses**							
0 (unvaccinated)	**2,435 (26)**	1,705 (29)	730 (22)	**430 (20)**	333 (22)	68 (16)	29 (11)
1	**780 (8)**	461 (8)	319 (10)	**186 (8)**	116 (8)	39 (9)	31 (12)
2	**2,655 (29)**	1,618 (27)	1,037 (31)	**605 (28)**	415 (27)	116 (28)	74 (29)
3	**1,843 (20)**	1,090 (18)	753 (23)	**552 (25)**	354 (23)	132 (31)	66 (26)
4	**878 (10)**	581 (10)	297 (9)	**271 (12)**	180 (12)	52 (12)	39 (15)
5	**438 (5)**	339 (6)	99 (3)	**102 (5)**	83 (5)	8 (2)	11 (4)
≥6	**193 (2)**	133 (2)	60 (2)	**53 (2)**	39 (3)	6 (1)	8 (3)

**Abbreviations:** HHS = U.S. Department of Health and Human Services; ICATT = Increasing Community Access to Testing program; NA = not applicable; NAAT = nucleic acid amplification test; SGT = spike gene target; SVI = Social Vulnerability Index.

* Tests included in the subanalysis represent a subset of those included in the full analysis.

^†^ Persons of Hispanic or Latino (Hispanic) origin might be of any race but are categorized as Hispanic; all racial groups are non-Hispanic.

^§^ Regions are defined by HHS and include only states and territories with ICATT sites. U.S. Virgin Islands (Region 2) and American Samoa, Federated States of Micronesia, Guam, Marshall Islands, Northern Mariana Islands, and Palau (Region 9) were not included because they did not have pharmacies participating in ICATT. https://www.hhs.gov/about/agencies/iea/regional-offices/index.html.

^¶^ SVI is a composite measure that uses U.S. Census Bureau data on 16 social factors to rank social vulnerability by U.S. Census Bureau tract. The scale is from 0 to 1; higher SVIs represent more vulnerable communities. Tests with missing SVI data (<1% of total) were excluded from all analyses. https://www.atsdr.cdc.gov/placeandhealth/svi/data_documentation_download.html

** Rapid NAAT was performed on-site on self-collected nasal swabs using ID Now (Abbott Diagnostics Scarborough, Inc.), Xpert Xpress (Cepheid), and Accula (Thermo Fisher Scientific).

^††^ Laboratory-based NAAT was performed on self-collected nasal swabs at contracted laboratories using a variety of testing platforms.

^§§^ Persons were assumed to have received only original monovalent COVID-19 vaccine doses if they reported receiving their last dose before September 2, 2022, or if they reported receiving 1 or 2 total doses before April 18, 2023; persons were assumed to have received a bivalent dose and no updated dose if they reported receiving >2 total doses with their last dose during September 2, 2022–April 18, 2023, or receiving any number of doses with their last dose during April 19–September 12, 2023; persons reporting receipt of a dose after September 12, 2023, were assumed to have received an updated dose because these were the only authorized COVID-19 doses in the United States. https://www.cdc.gov/vaccines/covid-19/clinical-considerations/interim-considerations-us-appendix.html

^¶¶^ “Updated” refers to 2023–2024 monovalent COVID-19 vaccine.

**TABLE 2:** Effectiveness of updated 2023–2024 monovalent COVID-19 vaccine against symptomatic SARS-CoV-2 infection among adults aged ≥18 years, by interval since last dose and age group — Increasing Community Access to Testing program, United States, September 2023–January 2024

Age group, yrs/COVID-19 vaccination dosage pattern	Total no. of tests	SARS-CoV-2–positive test results, no. (row %)	Median days (IQR) since last dose among vaccinated	VE* (95% CI)
**≥18**				
No updated dose (Ref)	**8,097**	3,014 (37)	670 (422–843)	Ref
Received updated dose	**1,125**	281 (25)	52 (29–75)	54 (46–60)
7–59 days earlier	**634**	140 (22)	32 (19–46)	58 (48–65)
60–119 days earlier	**491**	141 (29)	79 (68–90)	49 (36–58)
**18–49**				
No updated dose (Ref)	**6,475**	2,332 (36)	681 (429–852)	Ref
Received updated dose	**681**	150 (22)	53 (30–74)	57 (48–65)
7–59 days earlier	**381**	69 (18)	32 (19–46)	64 (53–73)
60–119 days earlier	**300**	81 (27)	77 (67–89)	48 (31–60)
**≥50**				
No updated dose (Ref)	**1,623**	682 (42)	583 (398–787)	Ref
Received updated dose	**444**	131 (30)	50 (29–77)	46 (31–58)
7–59 days earlier	**253**	71 (28)	32 (21–45)	45 (26–60)
60–119 days earlier	**191**	60 (31)	81 (70–91)	47 (24–62)

**Abbreviations:** Ref = referent group; VE = vaccine effectiveness.

* VE = (1 − adjusted odds ratio) x 100. Odds ratios were calculated using multivariable logistic regression, adjusting for age (as a continuous variable), gender, race and ethnicity, Social Vulnerability Index of the testing location (<0.5 versus ≥0.5), pharmacy contractor, underlying conditions (presence versus absence), U.S. Department of Health and Human Services region of testing location, and date of testing. Previous analyses from this platform included local SARS-CoV-2 incidence in regression models; however, this variable is no longer available since the end of the public health emergency declaration in May 2023.

### VE by SGTF Status

In the subanalysis, 679 tests with *S*-gene target results from eligible persons were available, including 258 (38%) exhibiting SGTF (likely JN.1 lineages) and 421 (62%) with SGTP (likely non-JN.1 lineages) (Table 3). Because of recent emergence of JN.1 in the United States, VE was imprecise for tests with SGTF during the 7–59 days after receipt of updated vaccine. VE during the 60–119 days since receipt of updated vaccine was 49% (95% CI = 19%–68%) for tests with SGTF (median interval since dose = 80 days) and 60% (95% CI = 35%–75%) for tests with SGTP (median interval since dose = 73 days).

**TABLE 3:** Effectiveness of updated 2023–2024 monovalent COVID-19 vaccine against symptomatic SARS-CoV-2 infection among adults aged ≥18 years with samples tested at a commercial laboratory with spike gene target testing available, by interval since last dose and spike gene target status (N = 2,199) — Increasing Community Access to Testing program, United States, October 2023–January 2024

COVID-19 vaccination dosage pattern	Total no. of tests N = 2,199	SARS-CoV-2–negative test results		SARS-CoV-2–positive test results (n = 679)					
				SGT presence (likely non-JN.1)			SGT failure (likely JN.1)		
		No. (row %) n = 1,520	Median (IQR) days since last dose among vaccinated	No. (row %) n = 421	Median (IQR) days since last dose among vaccinated	VE* (95% CI)	No. (row %) n = 258	Median (IQR) days since last dose among vaccinated	VE* (95% CI)
No updated dose (Ref)	1,972	1,346 (68)	637 (398–805)	398 (20)	672 (402–800)	Ref	228 (12)	674 (412–816)	Ref
Updated dose, 60–119 days earlier^†^	**227**	174 (77)	80 (69–90)	23 (10)	73 (68–82)	60 (35–75)	30 (13)	80 (69–90)	49 (19–68)

**Abbreviations:** Ref = referent group; SGT = spike gene target; VE = vaccine effectiveness.

* VE = (1 – adjusted odds ratio) x 100. Odds ratios were calculated using multivariable logistic regression, adjusting for age (as a continuous variable), gender, race and ethnicity, Social Vulnerability Index of the testing location (<0.5 versus ≥0.5), pharmacy contractor, underlying conditions (presence versus absence), U.S. Department of Health and Human Services region of testing location, and date of testing. Previous analyses from this platform included local SARS-CoV-2 incidence in regression models; however, this variable is no longer available since the end of the public health emergency declaration in May 2023.

^†^ Overall VE, regardless of time since dose, and VE during the 7–59 days since vaccination were not calculated for the subanalysis based on SGT presence or SGT failure. Because of the timing of JN.1 spread in the United States, JN.1 VE estimates would be inherently weighted as longer time since dose and non-JN.1 VE estimates as shorter time since dose, biasing overall estimates of VE by lineage. Similarly, because of the timing of JN.1 spread, statistical power for VE for JN.1 lineages during the 7–59 days after receipt of vaccination was limited.

### Secondary VE Analyses

Secondary analyses showed similar VE estimates for receipt of updated vaccine compared with those who previously received only original monovalent doses and those who received original monovalent and bivalent doses. (Supplementary Table; https://stacks.cdc.gov/view/cdc/145937).

## Discussion

This report provides early estimates of effectiveness of updated monovalent XBB.1.5 COVID-19 vaccines against symptomatic SARS-CoV-2 infection and the first estimates of VE against symptomatic infection with the JN.1 lineage. These preliminary estimates from pharmacy testing conducted during September 2023–January 2024 showed updated monovalent COVID-19 vaccine provided protection for JN.1 and other circulating lineages.

VE against symptomatic infection provides helpful information about the range of protection provided by updated vaccines and against emerging lineages. An important strength of ICATT SARS-CoV-2 testing data is the ability to distinguish JN.1 from XBB lineages, allowing for comparison of VE during the same period after vaccination. Monitoring the potential impact on VE of JN.1 is critical because of the spike mutations in JN.1 (as compared with XBB lineages), which might be associated with increased immune escape^§§§§^ (*5*). Recent laboratory data show that the updated vaccines elicit neutralizing antibodies against emerging XBB lineages and JN.1 (*6*). Although point estimates during the 60–119 days after vaccination were lower for SGTF than SGTP results in this analysis, CIs overlapped, indicating no statistically significant difference. These data provide reassurance that updated vaccines are providing protection against JN.1 and XBB lineages.

These early estimates include the period only through 119 days since vaccination, a relatively brief postvaccination period, with no substantial waning. Because consistent patterns of waning VE were observed after original monovalent and bivalent COVID-19 vaccination, waning of VE is expected with more time since updated vaccination, especially against less severe outcomes such as symptomatic infection. Additional analyses conducted at longer intervals since authorization of updated vaccines are needed for continued monitoring of expected waning and to determine how well vaccines are working to prevent severe disease.

### Limitations

The findings in this report are subject to at least five limitations. First, vaccination status, previous infection history, and underlying medical conditions were self-reported and might be subject to recall bias. Self-reported frequency of previous infections >90 days before testing differed by vaccination status and SGTF status, but statistical power was not adequate for stratification of results. Further, previous infection is likely underreported (*7*). Previous infection provides some protection against repeat infection (*8*) and U.S. adults have a high prevalence of infection-induced SARS-CoV-2 immunity.^¶¶¶¶^ Thus, VE in this analysis reflects the current situation among U.S. adults and can be interpreted as the incremental benefit of receipt of updated COVID-19 vaccine beyond existing vaccination-induced, infection-induced, or hybrid immunity. Second, test registration questionnaires did not ask registrants about the number of updated vaccine doses received; therefore, the analysis might have included some persons who received >1 updated dose. Third, these estimates are derived from a population choosing to be tested for SARS-CoV-2 and are potentially subject to selection biases related to these factors. In addition, updated vaccination coverage to date has been low (approximately 22% as of January 13, 2024*****) among persons aged ≥18 years and varies by age, which could bias results if persons being vaccinated earlier are systematically different from those vaccinated later. Thus, residual confounding might be present and could affect these early estimates. Fourth, this analysis used a subset of data with SGTF status as a proxy for infection with a JN.1 lineage. Although SGTF identifies other BA.2.86 lineage viruses, JN.1 represents the majority of these and was the primary lineage increasing in proportion during the analytic period. Finally, this analysis did not control for time since receipt of the most recent dose before the updated dose; however, because of waning effectiveness of previous doses, particularly against symptomatic infection^†††††^ (*9*), this limitation likely had a minimal effect on results.

### Implications for Public Health Practice

Updated monovalent COVID-19 vaccines provided 54% (95% CI = 46–60%) protection against symptomatic SARS-CoV-2 infection in persons recently vaccinated compared with those who did not receive an updated vaccine dose. Vaccination provided protection for infections caused by JN.1 and infections caused by XBB-related lineages. Waning of effectiveness is expected with additional elapsed time since vaccination, especially against less severe disease. CDC will continue to monitor trends in VE. All persons aged ≥6 months should stay up to date with COVID-19 vaccination, including receiving a dose of updated vaccine.

## Footnotes

*
https://www.cdc.gov/respiratory-viruses/whats-new/SARS-CoV-2-variant-JN.1.html

†
https://covid.cdc.gov/covid-data-tracker/#variant-proportions

§ XBB sublineages representing >1% of all sequenced variants include HV.1, JD.1.1, HK.3, JG.3, and EG.5 (last updated January 9, 2024).

¶ SGTF lineages are defined by the presence of a deletion at positions 69–70 in the spike protein.

** ICATT vendors also report data for tests administered to people with medical insurance. Tests for persons with and without health insurance are included in this analysis.

†† The Social Vulnerability Index (SVI) is a composite measure that uses U.S. Census Bureau data on 16 social factors to rank social vulnerability by U.S. Census Bureau tract. The scale is from 0 to 1; higher SVIs represent more vulnerable communities. Tests with missing SVI data (<1% of total) were excluded from all analyses. https://www.atsdr.cdc.gov/placeandhealth/svi/data_documentation_download.html

§§ At test registration, adults report information on COVID-19 vaccination history, current COVID-19–like illness symptoms, history of previous positive SARS-CoV-2 test results, and underlying medical conditions. At Walgreens, comprising 95% of tests meeting inclusion criteria, test registrants who reported receiving COVID-19 vaccines were asked to report the total number of doses received and for the most recent dose, the manufacturer and the date of receipt as part of test registration. At CVS Pharmacy, comprising 5% of tests meeting inclusion criteria, test registrants' vaccination status was ascertained from a visit with a nurse practitioner or physician associate.

¶¶ Test registration forms asked persons to report whether they had an immunocompromising condition and provided the following examples: immunocompromising medications, solid organ or blood stem cell transplant, HIV, or other immunocompromising conditions.

*** Persons aged ≥12 years without immunocompromise and receiving updated Novavax COVID-19 vaccination are recommended to receive 2 updated COVID-19 vaccine doses if previously unvaccinated and 1 updated dose if previously vaccinated with any COVID-19 vaccine. https://www.cdc.gov/vaccines/covid-19/clinical-considerations/interim-considerations-us-appendix.html

††† On May 12, 2023, CDC removed guidance for use of Janssen COVID-19 vaccine because the vaccine was no longer available in the United States. https://www.cdc.gov/vaccines/covid-19/clinical-considerations/interim-considerations-us-appendix.html

§§§ Tests from persons reporting a positive SARS-CoV-2 test result during the preceding 90 days were excluded to avoid analyzing multiple tests for the same illness episode or reinfections within a relatively short time frame. https://www.cdc.gov/coronavirus/2019-ncov/symptoms-testing/testing.html

¶¶¶ Persons were assumed to have received only original monovalent COVID-19 vaccine doses if they reported receiving their last dose before September 2, 2022, or if they reported receiving 1 or 2 total doses before April 18, 2023; persons were assumed to have received a bivalent dose and no updated monovalent dose if they reported receiving >2 total doses with their last dose during September 2, 2022–April 18, 2023, or reported receiving any number of doses with their last dose during April 19–September 12, 2023; persons reporting receipt of a dose after September 12, 2023, were assumed to have received an updated monovalent dose because these were the only authorized COVID-19 doses in the United States during that period. https://www.cdc.gov/vaccines/covid-19/clinical-considerations/interim-considerations-us-appendix.html

**** Multivariable logistic regression models were controlled for age (as a continuous variable), gender, race and ethnicity, SVI of the testing location (<0.5 versus ≥0.5), pharmacy contractor, underlying conditions (presence versus absence), U.S. Department of Health and Human Services region of testing location, and date of testing. The following underlying conditions were included on the test registration questionnaire: heart conditions, high blood pressure, overweight or obesity, diabetes, current or former smoker, kidney failure or end stage renal disease, cirrhosis of the liver, and chronic lung disease (such as chronic obstructive pulmonary disease, moderate to severe asthma, cystic fibrosis, or pulmonary embolism).

†††† 45 C.F.R. part 46.102(l)(2), 21 C.F.R. part 56; 42 U.S.C. Sect. 241(d); 5 U.S.C. Sect. 552a; 44 U.S.C. Sect. 3501 et seq.

§§§§ JN.1 is a sublineage of BA.2.86, defined by the spike substitution L455S. Changes at this amino acid position have conferred immune escape advantages to other lineages and might be associated with increased immune escape.

¶¶¶¶
https://covid.cdc.gov/covid-data-tracker/#nationwide-blood-donor-seroprevalence-2022

*****
https://www.cdc.gov/vaccines/imz-managers/coverage/covidvaxview/interactive/adult-coverage-vaccination.html

†††††
https://www.cdc.gov/vaccines/acip/meetings/downloads/slides-2023-09-12/05-COVID-Link-Gelles-508.pdf
